# Nasal carriage of methicillin-resistant *Staphylococcus aureus* (MRSA) at a palliative care unit: A prospective single service analysis

**DOI:** 10.1371/journal.pone.0188940

**Published:** 2017-12-11

**Authors:** Maria Heckel, Walter Geißdörfer, Franziska A. Herbst, Stephanie Stiel, Christoph Ostgathe, Christian Bogdan

**Affiliations:** 1 Department of Palliative Medicine, Comprehensive Cancer Center CCC Erlangen-EMN, Universitätsklinikum Erlangen, Friedrich-Alexander-Universität (FAU) Erlangen-Nürnberg, Germany; 2 Mikrobiologisches Institut - Klinische Mikrobiologie, Immunologie und Hygiene, Universitätsklinikum Erlangen and Friedrich-Alexander-Universität (FAU) Erlangen-Nürnberg, Germany; 3 Institute for General Practice, Hannover Medical School, Germany; Universitatsklinikum Hamburg-Eppendorf, GERMANY

## Abstract

**Background:**

The emergence of multidrug-resistant bacterial microorganisms is a particular challenge for the health care systems. Little is known about the occurrence of methicillin-resistant *Staphylococcus aureus* (MRSA) and multidrug-resistant Gram-negative bacteria (MDRGNB) in patients of palliative care units (PCU).

**Aim:**

The primary aim of this study was to determine the carriage of MRSA among patients of a PCU at a German University Hospital and to assess whether the positive cases would have been detected by a risk-factor-based screening-approach.

**Design:**

Between February 2014 and January 2015 patients from our PCU were tested for MRSA carriage within 48 hours following admission irrespective of pre-existing risk factors. In addition, risk factors for MRSA colonization were assessed. Samples from the nostrils and, if applicable, from pre-existing wounds were analysed by standardized culture-based laboratory techniques for the presence of MRSA and of other bacteria and fungi. Results from swabs taken prior to admission were also recorded if available.

**Results:**

297 out of 317 patients (93.7%) fulfilled one or more MRSA screening criteria. Swabs from 299 patients were tested. The detection rate was 2.1% for MRSA. All MRSA cases would have been detected by a risk-factor-based screening-approach. Considering the detected cases and the results from swabs taken prior to admission, 4.1% of the patients (n = 13) were diagnosed with MRSA and 4.1% with MDRGNB (n = 13), including two patients with MRSA and MDRGNB (0.6%). The rate of MRSA carriage in PCU patients (4.1%) was elevated compared to the rate seen in the general cohort of patients admitted to our University Hospital (2.7%).

**Conclusions:**

PCU patients have an increased risk to carry MRSA compared to other hospitalized patients. Although a risk factor-based screening is likely to detect all MRSA carriers amongst PCU patients, we rather recommend a universal screening to avoid the extra effort to identify the few risk factor-negative patients (<7%). As we did not perform a systematic MDRGNB screening, further studies are needed to determine the true prevalence of MDRGNB amongst PCU patients.

## Introduction

The worldwide emergence of multidrug-resistant bacterial microorganisms (MDROs) is a particular challenge for health care systems [1]. First, infections with MDROs are more difficult to treat, because only second-line antibiotics can be used. Second, patients that are colonized with a MDRO usually suffer from underlying comorbidities and have an increased risk to develop an infection with this bacterium. Moreover, these patients are a potential source for the transmission of MDROs within hospitals. Third, patients colonized or infected with MDROs frequently require special infection control precautions such as the separation from other patients in order to prevent further spreading of the MDROs. These infection control precautions are an extra burden for the patients themselves, their visitors and family members as well as for the hospital staff and cause additional costs [2].

Methicillin-resistant *Staphylococcus (S*.*) aureus* (MRSA) has been the classical MDRO which accounts for ca. 1% to more than 50% of all *S*. *aureus* infections, depending on the country and the clinical setting [3]. Vigorous measurements for identification and isolation of MRSA carriers have significantly helped to reduce the prevalence of MRSA in several countries. In Germany, the rate of MRSA strains among the recorded clinical *S*. *aureus* isolates went down from 21.4% in 2005 to 11.8% in 2014 [4]. Accordingly, the percentage and absolute number of nosocomial MRSA infections in Germany significantly decreased in a 6 year time-period [5].

A novel microbial threat in clinical medicine is the spreading of multidrug-resistant Gram-negative bacteria (MDRGNB), which comprises strains of *Pseudomonas aeruginosa*, *Acinetobacter baumannii* and various enterobacteria that have lost their susceptibility to major groups of antibiotics (group 3 and 4 cephalosporins [also termed third- and fourth-generation cephalosporins], carbapenems, fluoroquinolones) [6,7]. These bacteria are difficult to treat as only very few antibiotic therapeutic options exist that are mostly much less well tolerated [6,8]. In many countries, including Germany, measurements for prevention of infections with and spreading of MDROs have become a key priority in the health care system [9,10]. In Germany, the Commission for Hospital Hygiene and Infection Prevention (KRINKO) has recently launched updated guidelines for the management of MRSA and MDRGNB in medical and healthcare institutions [11,12]. These include recommendations for the active detection and isolation of patients colonized with MDROs using targeted or universal screening strategies as well as instructions for precautions during the nursing of those patients. In addition, Germany has implemented in 1995 a nationwide nosocomial infections surveillance system [13].

A scant body of current literature about MRSA in palliative and hospice care settings suggests that the prevalence rate might range from 3 to 11.6% [14]. Findings for MDROs other than MRSA have not yet been published. Palliative care and hospice patients are commonly assumed to have many risk factors, such as frequent hospital stay, indwelling catheters or the need for nursing care. Therefore, screening and detection measures appear to be indicated in these settings. The current literature, however, discusses different management options and does not advocate routine MDRO screening upon admission [19,20]. Taking into account that in end-of-life care the diagnosis of MRSA colonization and subsequent hygiene measurements might be stressful for the patients [21–26] and the best strategy for the handling of MDRO-positive patients in palliative care is a matter of ongoing debate, we aim to develop a patient-, family- and staff-centred approach for the management of MDRO colonization or infection of patients in in end-of-life care. The study presented here is part of a larger research project (“M-EndoL—MRSA in end-of-life care”) [27], which includes social, economic and regulatory aspects of the end-of -life situation and uses surveys of patients, family caregivers and staff members on satisfaction and quality of life and work.

### Study aim

The primary goal of this study was to determine the prevalence of MRSA colonization and/or infection among patients admitted to a German PCU using a universal admission screening. In an additional retrospective analysis, we also aimed to ascertain, whether the positive cases would have also been detected by the risk factor-based screening approach routinely applied in our university hospital. Swabs taken for the purpose of MRSA screening from nostrils and wounds were also analyzed for other bacteria as well as fungi. A systematic screening for MDRGNB (which would entail the analysis of additional body sites) was not performed.

## Methods

### Design of the study

The screening study took place in the PCU of the University Hospital of Erlangen, Germany, for the duration of twelve months between February 2014 und January 2015. The PCU holds ten beds and admits patients from (a) other clinical departments of the University Hospital Erlangen, (b) other hospitals inside or outside Erlangen, and (c) from nursing homes or other clinical settings for patient care. During this time-period, nasal and, if applicable, wound swabs were routinely taken from all patients admitted to the PCU within 48 hours following admission and analysed for MRSA, irrespective of screening criteria and risk factors (i.e. universal admission screening). Additionally, laboratory results from prior stays at the PCU as well as previous data from referring hospital wards were included in our analysis, i.e. (a) results from the risk-based screening in clinical departments (other than PCU) of the University Hospital Erlangen, available in the electronic hospital information system, and (b) results from non-systematic MDRO screening in hospitals and nursing homes outside Erlangen, as far as these data could be extracted from the previous medical reports.

### Definition of MDRGNB

Nasal swabs taken for MRSA screening were also analyzed for MDRGNB if the culture yielded the growth of Gram-negative bacteria. According to the recommendations of the German Commission for Hospital Hygiene and Infection Prevention (KRINKO) [11], we focused on enterobacteria, *Pseudomonas aeruginosa* and *Acinetobacter baumannii*, which have lost their susceptibility to 3 (termed in Germany "3MRGN") or 4 (termed in Germany "4MRGN") major groups of antibiotics (acylureidopenicillins; group 3 and 4 cephalosporins; carbapenems; fluoroquinolones), because these groups of MDRGNB requires special hygienic precautions even in a PCU setting. In contrast, enterobacteria with resistance to group 3 and 4 cephalosporins *without* combined resistance to fluoroquinolones or carbapenems were *not* screened for in this study, because PCU patients colonized with these bacteria do not require enhanced barrier nursing and sufficient options for antibiotic therapy still exist in these cases.

### Microbiological analyses

Swabs were taken from the nostrils and, if applicable, from pre-existing wounds using rayon swabs with Amies gel transport medium (Nerbe plus, Winsen, Germany). All swabs were examined by standardized microbiological laboratory techniques for the detection of bacteria and fungi [28]. MALDI-TOF mass spectrometry (Biotyper; Bruker Daltonik, Bremen, Germany) was used for species identification. Culture-based MRSA screening was performed using selective chromogenic media (CHROMagar™, MAST Diagnostica, Reinfeld, Germany) in addition to classical culture media (blood agar). CHROMagar™ shows high sensitivity for the detection of MRSA (>95%) and reduces detection time to 24 h in >95% compared to 48 to 72 h with blood agar [29,30]. MRSA was confirmed using an immunochromatographic PBP2a test (Alere, Scarborough, ME) or by PCR (GeneXpert system, Cepheid, Sunnyvale, CA) and subjected to antibiotic susceptibility testing using a VITEK 2 system (bioMérieux, Nürtingen, Germany). All discernible enterobacteria and non-fermenting bacteria grown on the blood agar plate were identified by MALDI-TOF mass spectrometry and also routinely tested for their antibiotic susceptibility (VITEK 2). The chosen procedures allowed a detailed microbiological characterization of the nasal swabs. Detection of MRSA or MDRGNB was immediately reported by phone to the PCU to initiate necessary hygiene measures or antibiotic treatment according to the guidelines of the University Hospital.

Ethical approval for the parent study including the routine screening procedure was obtained from the Ethics Committee of the Medical Faculty of the Friedrich-Alexander-University (FAU) Erlangen-Nürnberg (302_13 B, 15.01.2014).

### Further patient data collection and analysis

All admitted patients were also assessed whether they would have fulfilled the criteria of the national KRINKO commission [31,32] for risk factor-based screening that were implemented in modified form at the University Hospital Erlangen in 2008. According to these recommendations MRSA screening is carried out upon fulfilment of one or several of the following criteria:

1. known medical history of previous MRSA colonization or infection,
2. previous stay in an institution or region with high MRSA prevalence,
3. stay in a hospital for more than three days during the past 12 months,
4. (occupational) direct contact to farm animals (e.g. pigs), which have received antibiotics or where the use of antibiotics can be assumed based on the size of the farm,
5. previous contact to carriers of MRSA (e.g. shared room with a previously undetected MRSA carrier in the hospital),
6. patients with two or more of the following risk factors:
  - permanent need for nursing care assistance,
  - antibiotic therapy (≥ 5 days) during the past 6 months,
  - indwelling catheter (e.g. urinary bladder catheter, percutaneous endoscopic gastrostomy tube),
  - kidney disease requiring dialysis,
  - skin ulcers, gangrene, chronic skin wounds, deep soft tissue infections,
  - burn injuries.

A set of core data containing the personal data of the patients as well as the details on their care and disease, using the German Hospice and Palliative Care Evaluation (HOPE), was part of our standard documentation [33].

The program IBM SPSS Statistics 21 (SPSS Inc., Chicago, IL, USA) for Windows was used for statistical evaluation. Descriptive statistics with means, medians, minimum-maximum range, and standard deviations (SD) were calculated and frequency analyses were generated in order to describe the population and microbiological test results. Only anonymized data were saved and processed electronically.

## Results

### Screening rate

The study included 317 patients, from which 23 patients were re-admitted during the study period (19 patients with two stays each, three with three stays each, one with five stays). The study sample and the further analyses refer to the 317 patients and only to the first visit of readmitted patients.

Nasal swabs were taken from 299 patients (94.3%) as part of our routine diagnostic procedure; from 9 of these patients, additional swabs from pre-existing wounds were analysed (3.0%). The combined results of nasal and wound swabs are shown in Table 1. All specimens were subjected to microbiological cultures within a mean (± SD) interval of 1.4 (± 0.6) days after admission of the patient (range from 1 to 5 days).

**Table 1:** Overview on the presence of MDROs in the 317 patients admitted to the PCU.

Number of patients to be screened at the PCU			317
**Number of patients screened at the PCU**^a^	one swab from nasal atrium plus one swab from wound at admission in 9 cases		**299**
Positive for MDRO at the PCU			7
		MRSA pos.	5
		MDRGNB pos.	1
		MRSA+ MDRGNB pos.	1^c^
Negative for MDRO at the PCU^b^			276
	Previous positive result^d^		8
		MRSA pos.	0
		MDRGNB pos.	8
		MRSA+ MDRGNB pos.	0
Screening culture sterile			16
	Previous positive result^d^		1
		MRSA pos.	0
		MDRGNB pos.	1
		MRSA+ MDRGNB pos.	0
**Screening not performed at the PCU**			**18**
	Previous positive result^d^		8
		MRSA pos.	6
		MDRGNB pos.	1
		MRSA+ MDRGNB pos.	1
	Previous result^d^	negative	7
	No previous swab recorded		3

^a^ For patients that were readmitted, the screening result obtained during their first stay is recorded in this table.

^b^ The microbiological findings of the nasal swabs that were negative for MRSA and MDRGNB are given in Table 4.

^**c**^ MRSA detected in nasal swab, *Pseudomonas aeruginosa* (3MRGN) in wound swab

^**d**^ 97 of the 317 patients (30.6%) were swabbed prior to their stay at the PCU. The swab was taken 9.8 (± 7.6) days (range from 0 to 36 days; n = 96) before admission.

Collecting of nasal swabs was omitted in 18 cases (5.7%) because of a positive MDRO test prior to admission to the PCU (n = 8) or because of other reasons (n = 10). Other reasons included very short stays of the patients at the PCU due to their sudden death on the day of admission or the day thereafter (n = 7) or organizational losses (n = 3).

### Description of study sample

The mean age (± SD) of the patients (n = 317) was 70.6 (± 13.7) years (range from 27 to 99 years). Almost half of the patients (47%) were females (Table 2). The majority of patients were transferred from another medical unit of the University Hospital Erlangen (68.8%). 18.0% of the patients were admitted from their private home and 13.2% from other institutions. The mean (± SD) length of the stay at the PCU was 9.7 (± 7.2) days (range from 1 to 43 days). More than half of the study sample (60.6%) died at the PCU and 39.4% were discharged either to their private home (21.2%) or transferred to another institution or hospital (18.2%). More than two third of the patients (68.8%) were diagnosed with cancer. Most patients suffered from complex pain symptoms (38.2%) or neurological/ psychological symptoms (34.1%) at the time of admission (Table 2).

**Table 2:** Demographic and health related data.

**Patients at PCU (N = 317)**			
**Age (mean** ± **SD; range)**	70.6 (± 13.7) years (27–99 years)		
**Gender**	Male		53% (n = 150)
	Female		47% (n = 167)
**Diagnosis**	Cancer		68.8% (n = 218)
	Non-malignant disease		31.2% (n = 99)
**Duration of stay (mean** ±**SD; range)**	9.7 (±7.2) days (1–43 days)		
**End of treatment**	Discharge	39.4% (n = 125)	
		to private home	21.1% (n = 67)
		to hospice	11.0% (n = 35)
		to nursing home	1.6% (n = 5)
		to short-term care	1.9% (n = 6)
		to other hospital	2.2% (n = 7)
		to rehabilitation centre or other ward	1.3% (n = 4)
		not known	0.3% (n = 1)
	Death	60.6% (n = 192)	
**Symptom complexes (multiple answers)**	Pain		38.2% (n = 121)
	Neurological/psychological		34.1% (n = 108)
	Respiratory		30.3% (n = 96)
	Gastrointestinal		14.8% (n = 47)
	Wounds		4.1% (n = 13)
	Urogenital		3.5% (n = 11)
	Others		33.4% (n = 106)

### MRSA detection rate

From 317 patients admitted to the PCU, 299 were tested for MDROs taking swabs from the nostrils. 16 of the 299 nasal swabs remained sterile (see Table 1), indicating previous antibiotic treatments and/or insufficient sampling of the nostrils. In six cases the nasal swabs obtained after admission were newly positive for MRSA. Thus, the MRSA detection rate, which was calculated from the 282 nasal swabs yielding a positive culture, was 2.1%. Seven patients were already known to be positive for MRSA due to recent testing, and therefore were not screened again at the time of admission to the PCU. The overall prevalence rate for MRSA was therefore 4.1% (13 out of 317 patients).

### MRSA screening criteria and risk factors

In this study 297 patients (93.7%) fulfilled one or more criteria for performing the risk factor-based MRSA-screening implemented at our university hospital. Most often, patients had stayed in a hospital for more than three days during the past 12 months (81.1%), or they had a particular risk of contracting MRSA due to their permanent need for nursing care assistance (69.1%) or an indwelling catheter (41.6%) (Table 3).

**Table 3:** Recorded MRSA-screening criteria and MRSA risk factors (n = 317 patients).

**Screening Criteria**	
Stay in hospital >3 days (within last 12 months)	81.1% (n = 257)
Previous stay in an institution with high MRSA prevalence	8.2% (n = 26)
Medical history of previous MRSA colonization or infection	2.8% (n = 9)
(Occupational) direct contact to farm animals (e.g. pigs), which have received antibiotics	0.0% (n = 0)
None recorded	7.9% (n = 25)
**Risk factors** (multiple answers)	
Permanent need for nursing care assistance (SPI <32)^a^	69.1% (n = 219)
Use of indwelling catheter	41.6% (n = 132)
Antibiotic therapy (>5 days within last 6 months)	31.5% (n = 100)
Ulcers or chronic wounds	8.2% (n = 26)
Kidney disease requiring dialysis	1.9% (n = 6)
Burn injuries	0.0% (n = 0)
None recorded	15.1% (n = 48)

^a^The “Selbstpflegeindex (SPI)” is part of a German outcome-oriented nursing assessment and is meant to predict the risk of insufficient care after discharge from hospital. The SPI consists of ten items and its sum score ranges between 10 (= maximum impairment of self-care) and 40 (= full ability of self-care). The SPI is part of the routine documentation and the cut-off point is <32.

All patients who tested positive for MRSA fulfilled at least one criterion for the risk factor-based MRSA-screening routinely performed at our university hospital so that all these patients would have been subjected to the targeted screening procedure.

### Incidental detection of carriers of MDRGNB

In two patients the nasal swabs obtained after admission revealed a previously unknown colonization with MDRGNB. An additional group of 11 patients was known to be positive for MDRGNB based on the analysis of various clinical specimens (six urine samples, two wound swabs, two bile aspirates, one blood culture) (Table 1). While two of these 11 patients were not tested again at the time of admission, nasal swabs taken from 9 of these patients were negative for MDRGNB. This result clearly shows that nasal swabs are not sufficient for detecting MDRGNB carriers and that a reliable MDRGNB screening requires the analysis of additional specimens such as rectal swabs.

The 13 isolates of MDRGNB comprised three *Pseudomonas aeruginosa* (two 4MRGN, one 3MRGN), six *Escherichia coli* (all were 3MRGN and expressed extended-spectrum beta-lactamases [ESBL]), two *Klebsiella pneumoniae* (3MRGN with ESBL), one *Klebsiella oxytoca* (3MRGN with K1 beta-lactamase) and one *Enterobacter cloacae* (AmpC beta-lactamase). Their detailed antibiotic susceptibility profile is presented in S1 Table. Carbapenem-resistant enterobacteria were not detected. Overall, in 13 out of 317 patients (4.1%) MDRGNB were found, although a systematic screening had not been performed.

The microbiological analysis of the nasal swabs further revealed that even in the absence of MRSA or MDRGNB a high proportion of patients (81 of 291, 27.8%) showed a pathological nasal flora (e.g. predominant colonization with methicillin-susceptible *S*. *aureus*; presence of enterobacteria and/or fungi) (Table 4).

**Table 4:** Microbiological findings of the nasal swabs negative for MRSA and MDRGNB (n = 292).

Category	Total no. of nasal swabs	Specification	No. of swabs positive for the specified bacteria
**normal microbiological result**	195	normal nasal flora^a^	195 ^b^
**pathological microbiological result**	81^c^	predominant detection of *Staphylococcus aureus* (MSSA)	38
		predominant colonization with enterococci	2
		colonization with Streptococcus dysgalactiae subsp. equisimilis	1
		presence of Gram-negative facultative pathogenic bacteria	37
		*Citrobacter koseri*	2
		*Enterobacter aerogenes*	2
		*Enterobacter cloacae*	2
		*Morganella morganii*	1
		*Serratia marcescens*	1
		*Pseudomonas aeruginosa*	9
		*Stenotrophomonas maltophilia*	2
		non-differentiated non-fermentating Gram-negative bacteria	18
		predominant detection of fungi	19
		*Candida albicans* complex (*C*. *albicans/dublinensis*)	3
		other yeasts	14
		*Aspergillus fumigatus*	2
sterile after 2 days of culture	16		

^a^ e.g. α-hemolytic streptococci, coagulase-negative staphylococci, and corynebacteria.

^b^ Of the 195 swabs, 18 (9.2%) yielded low colonization with *S*.*aureus* (MSSA), 11 (5.6%) showed small quantities of *C*. *albicans* or other yeasts, and one (0.5%) was positive for *S*. *pneumoniae*

^c^ The total number of swabs with pathological results is smaller than the sum in the right column due to cases with 2 or 3 different cultured microbes.

### Characteristics of patients carrying MDRO

Patients admitted with positive MDRO findings (n = 24), either based on the PCU entrance screening (n = 7) or previous screenings (n = 17) (Table 1), were on average 71.5 years old (range 38 to 98 years, SD ±14.1 years) and not significantly different from patients without MDRO findings (T = -0.354, df = 315, p = 0.723). Almost half of them were female (n = 11). 41.7% (n = 10) of the patients suffered from non-malignant diseases and all patients showed low functional levels [34] (Eastern Cooperative Oncology Group performance status [ECOG] 3 [n = 5], ECOG 4 [n = 19]). Patients with positive MDRO findings were transferred to the PCU from a hospital (80.0%, n = 23) or their private home (8.0%, n = 2). Three patients were admitted to the PCU and stayed in a double bedroom before the positive MDRO finding was known. For one patient no contact precautions were undertaken, as the patient died shortly after the MDRO diagnosis.

15 patients did not have signs of acute infection. In five patients signs of infection were present that most likely were causally linked to the MRDO finding. Another four patients showed signs of infection which could not be clearly attributed to the detection of MDRO. More than half of the patients with positive MDRO finding had received antibiotic therapy (n = 14) prior to or during their stay at the PCU. In five patients carrying MRSA eradication measures (mupirocin nasal ointment, mouth rinsing with octenidin) were carried out (one of the patients received both antibiotic therapy and the eradication measures).

MDRO-positive patients stayed on average for 12.4 days (range 2 to 35 days, SD ±8.2 days) on the PCU ward, which was not significantly different from patients without MDRO findings (T = -1.909, df = 315, p = 0.057). Fifteen patients died at the PCU, nine patients were discharged to their private home (n = 6), to an inpatient hospice (n = 1), or to a nursing care home (n = 2).

## Discussion

In this study we present data from a systematic admittance screening for MRSA in a German specialist palliative care setting. The overall MRSA carriage rate for our collective of 317 patients, combining both newly detected cases (6 patients) and cases diagnosed prior to admission (7 patients), was 4.1%. In the available body of literature the prevalence rates ranged from 3.0 to 11.6% for MRSA in hospice and palliative care settings in different countries [14–17], including Germany [18]. However, the comparability of these data is limited because of differences in the screening policies (universal admission screening vs. risk factor-based screening), the frequency and quality of the screening procedure (e.g. number of omitted swabs, number of sterile swabs), the study populations (e.g. primary vs. repeated admissions of the patients), or the basic clinical setting (e.g., specialist palliative care setting, hospice, or palliative care service). Nevertheless, it is fair to argue that in our study the prevalence rate of MRSA amongst the patients admitted to our PCU was rather low (4.1%). Considering that almost all PCU patients fulfilled one or more of the MRSA screening criteria, the prevalence of MRSA was, as expected, higher compared to patients from acute care hospitals in Germany (2.2%) [35] and in the entire University Hospital Erlangen (2.7%, determined between October 2014 and May 2015: 54 positive out of 2012 nasal swabs; Geißdörfer, W., unpublished results).

In our study population of 317 patients MDRGNB were detected in 4.1% of the patients, although we did not perform a systematic screening and also did not apply procedures optimized for the detection of MDRGNB. In future studies on the prevalence of MDRGNB extranasal sites (e.g., rectal swabs or stool samples) need to be sampled, which are more suitable for the detection of colonizing MDRGNB. Furthermore, these studies should also specifically screen for ESBL-producing enterobacteria (with or without ciprofloxacin resistance) to allow for better comparability with international MDRGNB classification schemes.

Besides MDRO, we found a high proportion of patients with a pathological nasal flora reflecting again the morbidity of the patients in our study population. With 41.7% a disproportionately high number of patients with MDRO diagnosis were suffering from a non-malignant disease. Patients without cancer in palliative care are known to be older, in a lower functional status, afflicted more often by (chronic) wounds and have a higher need for nursing care [36]. Hence, the observed higher prevalence for MRSA reflects that PCU patients with chronic diseases are at a higher risk to acquire MDROs.

With respect to the frequently discussed issue of universal admission screening versus the risk factor-based screening strategy, it is important to bear in mind that the latter, if stringently applied, is likely to detect at least 80 to 90% of the MRSA carriers [35,37,38]. In addition, a universal admission screening is less cost-effective in most settings compared to the targeted risk factor-based screening [38–42]. A British study assessed different management systems in PCUs and hospices and reported that none of the 56 responding institutions routinely screened all in-patients at admission [19], even though they were considered as patients being at high risk for MRSA colonization or infection. However, in specialized palliative care a particular risk assessment seems dispensable considering that almost all admitted patients were positive for MRSA risk factors as illustrated by our study (>93%). Thus, a universal MRSA screening should be established at PCUs to avoid the additional effort necessary for evaluating risk factors and screening criteria in palliative care patients, of which ultimately only very few would be exempted from the screening.

Although polymerase chain reaction (PCR)-based assays with high negative predictive values (99.9%) are available and can detect MRSA in swabs within two hours, we opted against the use of this rapid MRSA testing in our PCU setting, for the following reasons. First, most palliative care patients are already admitted to single rooms, so that a rapid MRSA test result is not urgently needed. Second, the use of PCR would not replace an additional cultural approach, which is necessary for antibiotic susceptibility testing and exclusion of false-positive or false-negative PCR results (range of <1 to 4%, according to the manufacturers instructions and our own laboratory experience). Third, PCR-based assays would significantly increase the costs of the screening [43].

### Study limitations

A complete assessment of the entire list of MRSA-screening criteria and risk factors was not possible for all of our patients. Difficulties were encountered especially with patients that were transferred from out-patient care settings. Also, we did not succeed in obtaining detailed information from general practitioners, who previously treated the patient, on the prescription of antibiotics. Our study did not entail a systematic and methodologically appropriate screening for MDRGNB. Due to ethical considerations we refrained from taking swabs from locations other than the nostrils and pre-existing wounds, although rectal swabs and pre-enrichment methods are recommended for MDRGNB [11,44]. Therefore, we cannot draw firm conclusions on the actual prevalence rates of MDRGNB amongst PCU patients, which might be considerably higher than observed here.

It was not possible to swab all admitted patients due to organizational and situational factors. Furthermore, carriers with low MRSA loads might have been missed by the chosen screening procedure, because (a) rayon swabs used in our study showed a reduced recovery rate for MRSA as compared to flocked swabs [45,46] and (b) an enrichment procedure was omitted [47].

The data presented here were collected at a single PCU. Therefore, the results cannot be readily applied to other regions or settings.

## Conclusions

The results of this study advance our knowledge about the occurrence of MRSA in palliative care settings. The prevalence rate of MRSA in PCU patients is higher than in general acute hospital populations. Our findings show that patients transferred to the PCU bear a high risk for previous acquisition (colonization and/or infection) of MRSA (>93%). Therefore, universal MRSA-screening is recommended for reasons of simplicity. Although we incidentally also detected carriers of MDRGNB amongst our PCU patients, systematic studies are necessary before a routine MDRGNB screening (universal or risk factor-based) can be recommended. A positive screening result for MDRO has to lead to adequate hygienic measures. Adequacy in the context of end-of-life care implies to carefully weigh against each other protection and isolation requirements (e.g. for patients, relatives, visitors, health care workers) and the need of the palliative care patient to be socially included in his last phase of life.

## Supporting information

S1 Table Antibiotic susceptibility profiles of the obtained MDRGNB isolates. (DOCX)
