# Supplementary material for: Nasal carriage of methicillin-resistant Staphylococcus aureus (MRSA) at a palliative care unit: A prospective single service analysis
Source: PLoS One. 2017 Dec 11;12(12):e0188940. doi: 10.1371/journal.pone.0188940 (PMC5724845; doi:10.1371/journal.pone.0188940)
Supplement: S1 Table — (DOCX) [file pone.0188940.s001.docx]

S1 Table. Antibiotic susceptibility profiles of the obtained MDRGNB isolates

|  |  |  | Phenotype | | | | | |  |
| --- | --- | --- | --- | --- | --- | --- | --- | --- | --- |
| Species | Type of MDRGNB | Beta-lactamase^1^ | PIP | CRO | CAZ | FEP | MEM | CIP | Category^2^ |
| *P. aeruginosa* | 4MRGN | n.d. | i | n.d. | i | i | i | r | A |
| *P. aeruginosa* | 3MRGN | n.d. | r | n.d. | r | i | s | i | A |
| *P. aeruginosa* | 4MRGN | n.d. | r | n.d. | r | r | r | r | B |
| *E. coli* | 3MRGN | ESBL | r | r | r | n.d. | s | r | B |
| *E. cloacae* | 3MRGN | AmpC  (ESBL neg) | r | i | r | n.d. | s | i | C |
| *K. pneumoniae* | 3MRGN | ESBL | r | s | r | n.d. | s | i | C |
| *K. pneumoniae* | 3MRGN | ESBL | r | r | i | n.d. | s | r | C |
| *E. coli* | 3MRGN | ESBL | r | r | i | n.d. | s | r | C |
| *E. coli* | 3MRGN | ESBL | r | r | r | n.d. | s | r | C |
| *E. coli* | 3MRGN | ESBL | r | r | r | n.d. | s | r | C |
| *E. coli* | 3MRGN | ESBL | r | r | s | n.d. | s | r | C |
| *K. oxytoca* | 3MRGN | K1  (ESBL neg) | r | r | s | n.d. | s | i | C |
| *E. coli* | 3MRGN | ESBL | r | r | i | n.d. | s | r | C |

^1^ Beta-lactamases were phenotypically confirmed. ESBL-producing enterobacteria tested positive in the ESBL Etest^R^ using CTX/CTX-CLV and CAZ/CAZ-CLV combinations (bioMérieux, Marcy-l´Etoile, France). AmpC beta-lactamase in *E. cloacae* is characterized by resistance to penicillins, non-interpretable ESBL Etest^R^, and a negative FEP/CLV-double disc diffusion test (Jarlier V, Nicolas MH, Fournier G, Philippon A. Extended broad-spectrum beta-lactamases conferring transferable resistance to newer beta-lactam agents in *Enterobacteriaceae*: hospital prevalence and susceptibility patterns. Rev Infect Dis 1988; 10: 867-878). K1 beta-lactamase in *K. oxytoca* is characterized by negative ESBL Etest^R^, high level resistance to piperacillin/tazobactam and sensitivity to ceftazidime.

^2^ Categories: A, positive result at admission; B, previous positive result, no swab at admission; C, previous positive result, negative result at admission (see Table 1).

Abbreviations: PIP, piperacillin; CTX, cefotaxime; CRO, ceftriaxone; CAZ, ceftazidime; FEP, cefepime; CLV, clavulanate; MEM, meropenem; CIP, ciprofloxacin; r, resistant; i, intermediate susceptible; s, sensitive; n.d., not determined.
